# Multiple Autoimmune Complications After a Single Dose of Pembrolizumab

**DOI:** 10.7759/cureus.35871

**Published:** 2023-03-07

**Authors:** Hardeep S Ahdi, Sufyan Abdulmujeeb, Edward Nabrinsky

**Affiliations:** 1 Internal Medicine, Advocate Lutheran General Hospital, Park Ridge, USA; 2 Internal Medicine, Advocate Sherman Hospital, Elgin, USA; 3 Hematology/Oncology, Advocate Lutheran General Hospital, Park Ridge, USA

**Keywords:** hematology, immunology, oncology, pembrolizumab, immunotherapy

## Abstract

Pembrolizumab, a monoclonal antibody that inhibits programmed cell death protein-1 (PD-1), is an important treatment for various malignancies. Unfortunately, it has also been associated with a wide array of immune-related adverse events. We present a unique case of a patient who received a single dose of pembrolizumab and subsequently developed multiple immune-mediated complications, including dermatitis, hepatitis, myositis, myocarditis, and myasthenia gravis.

## Introduction

Targeted immunotherapies (e.g., pembrolizumab), which are directed against immune checkpoint modulators such as programmed cell death (PD-1), have become efficacious in the management and treatment for malignancies, including but not limited to renal cell carcinoma. Despite their markedly therapeutic effects, immune checkpoint inhibitors such as pembrolizumab can be associated with immune related adverse events related to increased immune activity [1]. Most immune-related adverse events are effectively treated with temporary discontinuation of the offending agent or by inducing temporary immunosuppression with agents such as corticosteroids or alternative immunosuppressants in refractory cases [2]. Here we highlight a case of a patient who developed multiple immune related adverse events after receiving a single dose of pembrolizumab. The purpose of this review is to highlight the different adverse events associated with pembrolizumab, as seen in our patient, and to promote increased awareness so that they can be identified promptly and started on appropriate therapy.

## Case presentation

A 58-year-old male with a history of non-alcoholic steatohepatitis and right radical nephrectomy due to metastatic renal cell carcinoma presented for evaluation of progressively worsening left-sided ptosis for four days prior to admission in addition to diffuse myalgias and generalized weakness. He completed one dose of pembrolizumab approximately 3-4 weeks prior due to metastatic disease. On arrival, his vital signs were stable and imaging studies were negative for an acute process. Laboratory results were notable for troponin peak of 2404 ng/L, with electrocardiogram non-suggestive of ischemic changes. His aspartate transaminase (AST) and alanine transaminase (ALT) peaked at 740 U/L and 681 U/L, respectively (baseline less than 100s) and creatine phosphokinase (CPK) peaked at 11205 U/L. Neurology, cardiology, and hematology/oncology were consulted. Myositis extended panel, voltage gated calcium channel, acetylcholine binding and blocking antibody (Ab), muscle-specific kinase Ab immunoglobulin (IgG) were unremarkable. Transthoracic echocardiogram was unremarkable. He was started on high dose steroids, intravenous immunoglobulin (IVIG) 667 mg/kg/d x 3 days for total 2g/kg, and pyridostigmine. His symptoms and liver function tests improved, and he was discharged home on oral prednisone. Unfortunately, within five weeks after pembrolizumab administration, he developed worsening thrombocytopenia and had a trough level of 12,000/ml by weeks 6-7. Haptoglobin, prothrombin/partial thromboplastin time, and fibrinogen were normal to rule out hemolysis and disseminated intravascular coagulation. Lactate dehydrogenase was elevated at 460 U/L. Due to concern for checkpoint inhibitor-induced immune thrombocytopenia refractory to high dose steroids, he completed a 5-day course of IVIG 0.5 mg/kg/day with gradual improvement in cell counts. He was also evaluated by dermatology and it was thought that his diffuse body rash was less likely related to prednisone therapy but instead related to a delayed onset reaction to pembrolizumab. He also had electromyography which revealed mild active myopathy of the upper extremities. Over time his troponin, CPK, and liver enzymes normalized over the span of one month.

## Discussion

Immune checkpoint inhibitors (ICIs) are a novel class of immunotherapy drugs that are an important part of treatment and management of various malignancies, particularly renal cell carcinoma. These monoclonal antibodies block inhibitory molecules, such as cytotoxic T-lymphocyte-associated protein 4 (CTLA-4), programmed cell death protein 1 (PD-1, e.g., pembrolizumab), or its ligand programmed cell death protein ligand 1 (PD-L1) expressed on T lymphocytes, antigen presenting cells and tumor cells stimulating an immune cell activation in the tumor microenvironment [3,4]. ICIs such as anti PD-1 agents including pembrolizumab have been approved for malignancies including but not limited to melanoma, non-small cell lung cancer, head and neck squamous cell carcinoma, renal cell carcinoma, and urothelial carcinoma [4]. PD-1 is a transmembrane receptor located on the surface of T lymphocytes and helps to inhibit T-cell activation. ICIs disinhibit T-cell activity by antagonizing the checkpoint molecules, causing a robust T-cell activation, and ultimately leading to enhanced anti-tumor immune-mediated response [5]. Due to their vast effect on T immune cells in a variety of tissues, ICIs are often associated with autoimmune related side effects which can affect nearly every organ causing varying degrees of severity including colitis, hepatitis, pneumonitis, hypothyroidism, autoimmune retinopathy, uveitis or iritis, rheumatic or neuromuscular related complications [3,6]. Although the spectrum of immunotherapy-related adverse events can be extensive and diverse, it remains unclear/controversial with ongoing studies evaluating timeline associated with these adverse events and why some side effects are predominant than others and why some patients experience it compared to others. Our case is a rare example of multiple toxicities encountered after a single dose of ICI therapy.

The overall incidence of neurological complications associated with immunotherapy ranges from 2-4%, with mild events (grades 1-2) occurring in up to 6-12% of patients and more serious events (grades 3-4) occurring in fewer than 1% with the usage of both anti-CTLA-4 or under anti-PD-1 therapy [6]. Grades 1-2 are defined as nonspecific neurological symptoms, including headaches, dizziness, paresthesia, or small fiber sensory neuropathies. Grades 3-4 are defined as myasthenia gravis (MG), autoimmune encephalitis, hypophysitis, multiple sclerosis. One study reported neurological complications associated with PD-1 antibodies where 2.9% (10/347) of patients developed neuromuscular complications after a median of 5.5 cycles of treatment [7]. Current literature review suggests that pembrolizumab has been associated with 7-9 de novo cases of MG, only a portion of which had associated concurrent CPK elevation [1,8]. One case report demonstrated that the patient developed MG-related symptoms after a second dose of pembrolizumab and was successfully treated with systemic corticosteroid therapy. MG is an autoimmune phenomenon characterized by fluctuating muscle weakness involving ocular, bulbar, respiratory and limb muscles [9]. MG is diagnosed based on typical symptoms and positive serum antibodies. Anti-acetylcholine receptor antibodies and anti-muscle specific kinase antibodies have high specificity for MG, both of which are found in 80-85% and 5-10% of patients with idiopathic MG [1]. However, false negative results are common with double seronegative cases, as seen in our patient. Multiple reports have suggested that symptoms can occur after multiple pembrolizumab doses; however, very few case reports have demonstrated MG symptoms after only a single dose, which could create a challenging dilemma when trying to investigate a possible correlation [10]. As seen in our patient, despite the myositis extended panel, voltage gated calcium channel, acetylcholine binding and blocking Ab, and muscle-specific kinase Ab IgG being unremarkable, patient was suspected to have seronegative MG and responded well to high dose steroids, IVIG, and pyridostigmine therapy.

Liver enzyme elevations are common in patients treated with ICI therapy. For example, mild to moderate serum aminotransferase elevations can occur up to 20-30% during pembrolizumab therapy and are usually self-limiting [11]. However, only about 1-4% of patients typically present with aminotransferase elevations up to 5X the upper limit of normal, which would involve temporary discontinuation of therapy. Rarely, 1-2% of patients can evolve into an immune mediated liver injury that can become severe enough to warrant corticosteroid therapy. The pattern of liver injury associated with immunotoxicity appears to show a hepatocellular or mixed pattern distribution, which appears to be consistent with other reports [11,12]. Our patient had a hepatocellular pattern predominance with elevated AST and ALT without any concerns for impaired synthetic liver function abnormalities. The onset of injury is usually after 2-4 cycles (1-3 months) after initiation of therapy. Studies have shown that ICI-mediated hepatotoxicity has been associated with other organ immunotoxicities. Depending on the severity of the liver injury, appropriate management entails initiation of high dose steroids, which are tapered over the course of at least 4-6 weeks [13]. Most patients with immune related hepatitis respond to corticosteroids (as seen in our patient who was already getting treated for suspected MG), but a substantial fraction require treatment with a secondary immunosuppressive agent.

**Figure 1:**
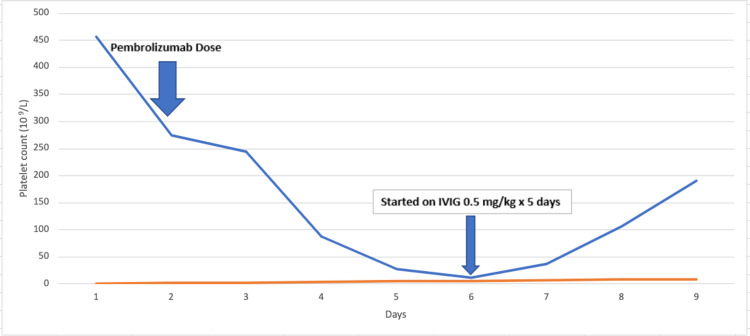
Platelet Trend after Pembrolizumab Initiation 1: Plt 457 (k/mcl) on 8/20; Pembrolizumab Dose given on 9/20; 2: Plt 275 on 9/21; 3: Plt 244 on 10/19; 4: Plt 88 on 10/29; 5: Plt 27 on 11/3; 6: Plt 12 on 11/6; 7: Plt 37 on 11/10; 8: Plt 107 on 11/21; 9: Plt 191 on 11/29 The X-axis denotes number of days that passed since beginning of treatment, while the Y axis denote platelet count.

Severe thrombocytopenia associated with ICIs is rare, with an incidence rate of about 0.8% [14]. There are currently 12 reported causes of ICI-related thrombocytopenia per literature review. Cytotoxic chemotherapy-related bone marrow toxicity is the leading cause of central thrombocytopenia in cancer patients [15]. However, the increasing number of immune checkpoint inhibitors have largely played a role and are likely to be associated with auto-immune cytopenia, suggesting that the spectrum of immune related events due to ICIs now extends to immune thrombocytopenia. Most cases were attributed to another ICI (e.g., nivolumab), and very few cases have been associated with pembrolizumab. In a case report by Mouri et al., a patient was found to have grade 4 thrombocytopenia with platelet (Plt) level 0.4 x 10^9^, 21 days following treatment with pembrolizumab and responded well to oral steroids (1 mg/kg/day) [16]. In another case studied by Song and Zhang, a patient was started on eltrombopag for severe refractory thrombocytopenia caused by pembrolizumab that was not responsive to high dose steroids combined with sufficient immunoglobulin therapy with obvious bleeding symptoms [14]. In our patient, as shown in Figure 1, after pembrolizumab was initiated his Plt count troughed approximately 47 days later, at which point he had been on an oral steroid taper. Given that his platelet count continued to decrease despite immunosuppressive therapy with steroids, he was started on a dose-adjusted IVIG 0.5 mg/kg daily for five days with gradual improvement in levels.

## Conclusions

In summary, autoimmune conditions caused by immunotherapies have been well documented in literature, whereas only a few cases where multiple toxicities were seen after a single dose are reported. Our case sheds some light on the uniqueness of up to four autoimmune related toxicities within a short time frame in the setting of a single dose of pembrolizumab. Immune-mediated adverse events typically occur within the first three months but can also occur as early as one day after initiation. In our patient, because workup for alternative etiologies causing his abnormal labs was unrevealing, it was believed that his symptoms and abnormal labs were due to pembrolizumab-induced immune-mediated response. Therefore, awareness of autoimmune-related complications with immune checkpoint inhibitors is advised, especially in the early phase of the treatment.
